# DEMENTIA CAREGIVER EMBRACE OF HOME VIDEO TELEHEALTH VISITS

**DOI:** 10.1093/geroni/igz038.911

**Published:** 2019-11-08

**Authors:** Lauren Moo

**Affiliations:** Geriatric Research Education & Clinical Center, Bedford, Massachusetts, United States

## Abstract

Bringing people with dementia to in-person medical visits can be logistically challenging for family caregivers, especially when they themselves are older adults with their own health or mobility challenges, when they live far from the clinic, or when they have to combat inclement weather. Our dementia management clinic has successfully trialed video visits into the home. Video sessions have been welcomed by many dementia caregivers citing reduced travel and less disruption of daily routine as the primary benefits of participating. Caregivers report equivalent visit satisfaction compared to in-person visits. While technical issues have been common, most were just brief audio or video lags. Expansion of HIPAA compliant telemedicine software options across devices is increasing the population of caregivers who are able to participate in home video visits. (127 words)

